# “The emperor’s new clothes”: a qualitative study of end users’ experiences from a failed large-scale implementation of an electronic health record system

**DOI:** 10.1186/s12913-026-14991-4

**Published:** 2026-06-23

**Authors:** Petra Dannapfel, Annie Axelsson, Susanne M. K. Gustavsson

**Affiliations:** 1 R&D Department, SV Hospital Group, Region Västra Götaland, Gothenburg, Sweden; 2 Department of Health, Medicine and Caring Sciences, Linköping University, Linköping, Sweden; 3 School of Health Sciences, University of Skövde, Post Box 408, Skövde, SE-541 28 Sweden; 4 Skaraborg Hospital, Lövängsvägen, Skövde SE-541 42 Sweden; 5 Department of Healthcare Development and Digitalization, Strategic Health, Västra Götalandsregionen, Gothenburg, Sweden

**Keywords:** Implementation, Organizational readiness, Electronic health record

## Abstract

**Background:**

Electronic health records (EHRs) play a vital role in healthcare organizations to manage patients’ health information. Implementing or transitioning to EHRs is often a large-scale implementation with many challenges. Organizational and individual readiness is crucial for successful adoption and implementation of an EHR system. The objective of this study was to explore clinicians’ and administrative staff’s experiences of a large-scale EHR implementation (Millennium) that was terminated shortly after its initial deployment.

**Methods:**

A qualitative design involving individual online interviews with 73 clinicians and staff investigated end users’ experiences after a large-scale implementation of EHRs in Sweden. A semi-structured interview guide was used during the interviews and included broad questions on individual and collective readiness to change before, during and after the implementation. Interviews were recorded and transcribed. The data were analysed using reflexive thematic analysis.

**Results:**

The findings show that experiences from a failed implementation highlight inadequate communication with the management level, inadequate preparedness in understanding the change and education on how to use the system. The results also show that the end users felt deceived, disappointed and overwhelmed with stress. End users highlighted the need to be involved in the implementation process and understand the whole picture. Lack of dialogue and feedback between co-workers and top management negatively affected organizational and individual readiness. Incomplete readiness and system contributed to anxiety, feelings of abandonment and being in an abstract reality.

**Conclusions:**

The findings provide an empirically grounded account of a failed large-scale implementation, reinforcing and extending key insights from implementation science. The study underscores that implementation is inherently a socio-organizational process, where meaningful end-user involvement and continuous, bidirectional communication between frontline staff and management are not peripheral but constitutive conditions for successful change. Anchored in Organizational Readiness for Change, the findings illustrate how limited readiness, combined with misalignment between system design and local context, can drive non-adoption and rapid abandonment. The system’s positioning as a comprehensive, modern solution raised expectations that were not met in practice, resulting in a sense of emptiness and disappointment among the participants, echoing the tale of the emperor’s new clothes.

**Supplementary Information:**

The online version contains supplementary material available at 10.1186/s12913-026-14991-4.

## Background

Management of big data and vast amounts of health information is required in healthcare organizations in this digital era. Electronic health records (EHRs) are crucial for storing, managing, and exchanging health information [1–3]. Expectations of EHR systems include increased efficiency, benefiting production, providing accurate, up-to-date, and comprehensive information for patient care, facilitating adherence to clinical guidelines and improving communication with patients and other providers [4, 5]. Benefits to patients, healthcare professionals, organizations, and the broader population are expected by improving the overall quality of care and patient safety [3, 4, 6]. However, despite significant investments in transitions to EHRs, many healthcare organizations struggle to ensure optimal use of the technology [7]. Some organizations have implemented EHRs in a reactive mode, fending off problems, rather than a proactive mode realizing benefits [7].

Healthcare organizations in high-income countries have faced problems when replacing their first-generation EHRs with large-scale suites (e.g. Millennium [Oracle] and Epic) [5]. Large-scale implementations of EHRs can be highly disruptive, based on their inherent complexity regarding the management of a large project, change management and acceptance, software solution design and realization of benefit [3, 8–10]. In addition, these transitions require coordination and collaboration among multiple stakeholders, whose diverse and evolving needs must be managed throughout implementation [3].

However, implementation theories may provide guidance for transitions to EHRs. Within the framework of implementation theory, it is posited that several determinants, including the characteristics of an innovation, are instrumental in determining the outcomes of implementation efforts [11]: the characteristics of an innovation (e.g. the complexity of the EHR system); the individuals who implement the innovation (e.g. attitudes towards the EHR system among staff); contextual factors (e.g. financial resources); and the strategies used (e.g. training offered to staff) [11]. These determinants for implementation act on multiple levels of an organization, interact and together contribute to implementation success or failure. Implementation is inherently a social process, deeply embedded within and inseparable from the context in which it occurs [12]. The Consolidated Framework for Implementation Research (CFIR) comprises five domains (intervention characteristics, outer setting, inner setting, characteristics of individuals, and process), all of which contribute to understanding and predicting the barriers and facilitators that influence implementation outcomes [13, 14]. The fifth domain concerns the implementation process, which typically involves an active and multi-level change effort to achieve effective use of the intervention as intended. This process is often driven by actors from both the inner and outer setting (e.g. local champions or external change agents) [15]. Further, it is the individuals in the organization who evaluate the implementation object, meaning that experts may perceive that the intervention meets the appropriate needs, whereas stakeholders within the organization may hold a different view. The domain of inner setting readiness for implementation is highlighted. Organizational readiness needs to be high to handle the complexity of large-scale implementations, such as implementing an EHR system in a healthcare setting [14].

Achieving organizational readiness for change is considered a crucial pre-implementation factor, and empirical studies indicate that the readiness for change is central for subsequent implementation processes and outcomes [2, 16]. According to Weiner [17], readiness is conceptualized as “the collective commitment and efficacy of all staff to implement the change”. Weiner [17] emphasizes the importance of the collective capability to execute the change and that readiness must be present at the individual, group, unit, department, and organizational level. Implementing complex organizational changes involves collective behaviour change and coordinating actions among many individuals, groups and sometimes also organizations.

In addition to the importance of readiness, there are several critical components involved in the implementation of EHRs, such as early testing, adaptation of the system to the larger organization, adaptation of the organization to the new system and the impact on the employees’ ability to perform their work duties in accordance with their roles and responsibilities [8, 18]. Challenges with implementing EHRs have been explained by several authors [3, 5, 19–22]. Several strategies and factors are critical for managing successful implementation of EHRs. For example, effective change and project management, adaptive leadership and governance, stakeholder engagement and meaningful clinician involvement where healthcare professionals can buy into the proposed benefits are crucial [4, 16]. Vanderhout et al. [4] highlight that assessing organizational readiness is valuable. Rigid top-down implementations often face resistance, whereas hybrid “middle-out” approaches tend to facilitate smoother transitions [3]. Hence, healthcare organizations often struggle to reap the benefits and achieve user satisfaction for several years after the implementation of EHRs [4, 5, 7].

In addition, the frequency of large-scale EHR implementations is increasing, highlighting the need for more knowledge to understand the specific challenges and characteristics of these large-scale and complex processes. Earlier research describes challenges with the implementation of large-scale her systems. However, studies on experiences from interrupted implementations are rare. In an example from Hawaii, an implementation was stopped in favour of a new EHR system better able to meet the needs of the hospital [18]. The objective of this study was to explore clinicians’ and administrative staff’s experiences of a large-scale EHR implementation (Millennium) that was terminated shortly after its initial deployment.

### Setting

This study was conducted in a Swedish healthcare organization in Region Västra Götaland (VGR), which includes five hospital groups (publicly funded), 200 primary care centres (public and private), and approximately 45,000 employees. The implementation was planned to be extensive and included healthcare in VGR and municipal healthcare in the region. The EHR systems in VGR were outdated and did not communicate with each other; therefore, there was a need to transition to a modern system. In 2017, Cerner’s (now Oracle) Millennium system was selected. The aim was to create a modern healthcare information environment to provide residents with accessible, high-quality healthcare and good involvement. The EHR system was supposed to be shared across the region’s primary care and hospital services, with access for municipal healthcare and private healthcare professionals and, importantly, the patients. In 2018, extensive work began to design a Swedish version of Millennium, focusing on gathering healthcare information around individual patients, which required clear regional standardization; approximately 2000 clinically active healthcare employees participated.

The introduction of Millennium meant major changes in workflows; therefore, there was a need for supportive regional and local change management structures. Each setting had its own local implementation project team, and they collaborated, led by the regional Millennium programme. Local support during Go Live (meaning the days of launching the EHR system live) consisted of super-users (Millennium coaches) and “floorwalkers” from Oracle. An IT support organization was reachable by telephone, chat or digital support. The timeline for Go Live was moved from 2021 to spring 2022 and then to fall 2024. The operational start was planned to take 2 years before all parts were up and running. The first Go Live started in the southern area of the region on November 12, 2024. The municipalities did not consider themselves prepared for the start and chose to wait. However, one hospital, several outpatient clinics and the regional lab organization did start.

Several problems arose on the first day, not all of which could be solved, and the actions taken did not have sufficient effect. There were technical problems, handling problems and patient safety was threatened. On November 15, it was decided to pause the implementation and return to the old systems, which happened gradually until November 20. The many reasons for the interruption were complex.

## Methods

A qualitative research approach with reflexive thematic analysis (RTA) [23] was used to explore clinicians’ and staff members’ experiences of the implementation of Millenium, a large-scale EHR system, which was terminated shortly after its initial deployment.

### Participants

Participants were healthcare professionals and staff members. Purposive sampling [24] was utilized to capture a wide range of professional roles and perspectives. The inclusion criterion was healthcare professionals and staff who were actively engaged with Millennium and working during Go Live. Seventy-three respondents participated. The participants’ characteristics are presented in Table 1. The number of participants per organization was based on their size; the target was 50 participants at the hospital, 20 participants in primary care and 10 participants in specialist outpatient clinics. The intention was to be able to listen to a broad range of perspectives of employees involved in the Go Live stage of the implementation.

**Table 1:** Characteristics of the participants (*N* = 73)

	Number (%)
**Gender (female)**	57 (78)
Age (years), mean (range)	43 (25–65)
**Professional occupation**	
Nurse	25 (35)
Physician	17 (23)
Assistant nurse	4 (5)
Other patient-oriented staff	9 (12)
Administration staff	18 (25)
**Organization**	
Hospital	47 (64)
Primary care	16 (22)
Specialist outpatient clinics	10 (14)
**How digital are you?**	
A. Mostly at work: primarily work‑focused digital use; minimal and intentional private use	3 (4)
B. Full‑time consumer: broad, practical digital use at work and in leisure; able to disconnect easily	39 (53)
C. All‑in: extensive, continuous digital engagement for tasks, communication, creation, and learning	31 (43)
D. Almost never outside work: very limited private digital use; digital activity mainly tied to work	0 (0)

Eligible individuals (*n* = 80) were invited by e-mail with written information about the study. Informed consent was obtained, and the participants were informed that participation was confidential and voluntary and that they could withdraw at any time during or after the interview. No compensation was given to the respondents, and no relationship was established between the interviewer and participants before the study. The interviews took place during respondents’ work time.

### Data collection

Data were collected through semi-structured interviews 4 months after Go Live of Millennium (i.e. after the EHR system went live). A semi-structured interview guide was used (Appendix 1) to capture experiences from (1) before Go Live, (2) during Go Live and (3) post implementation. It was used flexibly, allowing the interviewer to follow participants’ narratives with questions as they were discussed during the interview and remaining topics were covered before leaving the interview. Data collection was done by the authors (PD, AA, SG) and six other professionals with experience in interviewing.

Interviews were conducted digitally (Microsoft Teams), recorded, and the audio files were transcribed verbatim, resulting in approximately 1287 pages of transcribed material. The interviews lasted on average 46 min (range, 21–67 min). The interview guide was developed during a seminar with researchers and other professionals with experience in implementations to determine its ability to capture data relevant to the study aims. A pilot interview took place, and the questions were perceived to be informative and valid; thus, no revisions were made, and the pilot interview was included in the analysis.

### Data analysis

The data were analysed inductively using RTA [23]. RTA was chosen to enable the flexible yet systematic exploration of this large dataset, acknowledging the active role of the researcher in identifying patterns and aggregated meaning and meaningfulness across the data. The analysis process was performed by the three authors with a collaborative and reflexive team approach, aiming to develop a rich and more nuanced interpretation of the large dataset [25]. The authors’ pre-understanding comes from clinical experiences as nurses (SG and AA) and a behaviour scientist (PD). The first author has experience from earlier research on digitalization in health care. During the implementation of Millennium, PD was responsible for the implementation in one of the hospitals in VGR. However, the implementation there was planned for a few years later. The second author has clinical experience as a hospital nurse and in facilitating the implementation of digital processes. During the implementation of Millennium, the third author (SG) was first responsible for the implementation at a hospital in VGR, and later worked at the corporate office in VGR, with responsibility for collecting employees’ experiences of the Millennium implementation. The third author also has experience from research in quality science in healthcare. However, the authors and the other interviewers did not have any relationship with the interviewees before the interviews. To ensure careful consideration of the research perspective as well as other influences in the ongoing debate, the research team engaged in reflexive discussions throughout the analysis process. These discussions took place in numerous meetings in-person and online, for approximately 6 months.

Patterns of meaning were identified from the data through six phases as described by Braun and Clarke [23, 25]: (1) becoming familiar with the data; (2) coding; (3) generating initial themes; (4) developing and reviewing themes; (5) refining, defining, and naming themes; and (6) writing the report. In this study, familiarization was achieved by all authors (PD, AA, SG) by reading the transcribed interviews. The initial coding scheme was developed systematically, initially individually and subsequently in a series of workshops conducted with the three authors. First, each author labelled and coded the data from a third of the interviews. The codes were compared and discussed within the group to refine the codes and clarify their meaning. The authors went back to the dataset for new coding. In the next step, the codes and data were read by the group at a new workshop, compared, and interpreted to identify patterns of aggregated meaning and meaningfulness in the data, which led to the development of initial themes. The themes were then developed and reviewed by the authors both as a group and individually. By going back and forth in the data individually and in the group, the themes and codes were thoroughly discussed, refined, and defined to develop the final themes. The procedures for implementing each phase of this analysis are presented in Table 2.

**Table 2:** Description of the different phases of the data analysis process using reflective thematic analysis

The six phases of reflective thematic analysis [15]	Description of the process
1. Familiarize yourself with the dataset	Familiarization was achieved by all authors reading the entire transcribed interviews
2. Coding	The initial codes were developed systematically, first individually and then through several workshops involving the three authors. First, the authors individually labelled and coded the data in one third of the interviews each. The codes were compared and discussed within the group of authors to refine the codes and clarify their meaning. The authors went back to the dataset for new coding
3. Generating initial themes	The initial themes were developed when step codes and data were read by the group in a new workshop, compared, and interpreted to identify patterns of aggregated meaning and meaningfulness in the data that led to the development of initial themes
4. Developing and reviewing themes	The themes were developed and reviewed by the authors both as a group and individually
5. Refining, defining and naming themes	The data were reviewed repeatedly individually and in the group; the themes and codes were thoroughly discussed, refined, and defined to develop the final themes
6. Writing the report of the analysis	The results of the analysis were compiled and are reported in this article

## Results

The results were categorized into three phases: preparation phase, during Go Live and post implementation. Twelve themes are presented in Table 3. The themes are reinforced by quotes in the text (translated by the authors from Swedish into English).

**Table 3:** The themes identified from the interviews at each phase of implementing Millennium

Preparation phase	During Go Live	Post implementation
High expectations in the beginning	A big disappointment	Exhaustion and feelings of fatigue
Accelerated anxiety	Working in an abstract reality	Broken trust and lack of accountability
Difficult to understand the big picture	Insufficient local support	Conditional optimism about future implementations
Unsatisfactory communication	A minimum of response	
Variation in management support		

### Preparation phase

#### High expectations in the beginning

The need for a new EHR was expressed in different ways. Most respondents emphasized the importance of a new system and had high expectations early in the preparation phase. However, some felt their current system worked well. Expectations were that Millennium would support their work, save time, reduce administrative tasks, and allow for more patient interaction. There were high expectations regarding access to a comprehensive and continuous medical history in a single system.

In the beginning, you had very high hopes that this would be great and you were very positive about being able to get a more efficient system, because at first, there was a lot of talk about how it would be time-saving and more efficient, and there would be less documentation and less administrative time, so you had very high expectations. (Other patient-oriented staff 25)

Some respondents respected the scale and complexity of the transformation, noting that it is easier to maintain familiar routines than to undergo major change. They emphasized the importance of involving clinicians in the process and described themselves and their units as open to change. Implementation of Millennium was met with positive expectations and strong engagement, driven by a desire to contribute to successful outcomes.

#### Accelerated anxiety

The respondents said that there was some hesitation at the time about procuring an American system because working methods differ between American and Swedish healthcare. Expectations decreased, and users became more worried closer to Go Live. Many felt their concerns were dismissed and that management did not take their expertise seriously. Several groups, mainly physicians, became clearly critical before Go Live.

We are highly educated, with many years of college behind us. It’s not that we’ve been sitting around shouting just because we want to make a fuss, but it was clear early on that it wouldn’t work. (Nurse 9)

However, there were those who, despite the negativity, tried to maintain a positive attitude and did their utmost to make it go well.

This feels so-so, but I have to do my best given the situation. (Nurse 14)

As understanding of the system grew, so did anxiety about its readiness and the scale of the change. Respondents’ concerns about patient safety were prominent, especially regarding handling medications and referrals. Respondents felt reassured initially, but as unresolved issues persisted, stress and anxiety increased.

There was hope all along that this would be resolved, and we had been assured that it would, but when we were in the last week, it was still bugging us, and we still hadn’t received information about what would apply and how we would deal with things. Then things started to get really worrying, so the stress and anxiety over this dire situation increased again. (Physician 32)

#### Difficult to understand the big picture

This theme describes how different parts of the work and communication did not connect, which made it difficult to see and understand the big picture of Millennium’s functionality and the implementation process.

Early involvement of subject matter experts revealed difficulties envisioning the system’s functionality in a production environment because their contributions were limited to isolated components. The design process was further complicated by the extensive use of English, which posed additional challenges according to the respondents in understanding and contextualizing the system.

You needed quite a bit of visualization skill to be able to see how this works, and not everyone has that … was greatly limited by not having the underlying logic. (Nurse 19)

The respondents experienced that the insufficient testing of workflows and working methods before implementation complicated the rollout. Feedback after testing was lacking. Respondents questioned whether Millennium had been properly tested, noting minimal clinical involvement and a focus on technical components over user-centred workflows. Testing was experienced as fragmented and did not reflect the system as a whole.

There wasn’t much information about the system itself and what it looked like and how it was structured; rather, it was more of a revival meeting where they talked about how good this would be, how well it would work. (Administration staff 20)

Before implementation, the system was not fully developed, contextually adapted, or cohesively tested. Respondents reported limited opportunities to explore the system, which was perceived as fragmented, too generic, without clear coordination and difficult to navigate. It was difficult to understand the big picture; parts of the system worked well, but not as a whole.

Training in the system was primarily self-directed e-learning; materials included lengthy texts and complex manuals. The training environment was often experienced as malfunctioning and did not reflect the final system. Training was widely perceived as inadequate, incomplete, and poorly tailored to specific professional roles and did not fulfil expectations.

… the training itself was not as we had wanted, which made the expectations drop even more… it just became more and more frustrating. (Administration staff 40)

The content was not adapted to specific work settings, and participants received irrelevant information without essential guidance, and training did not provide enough opportunity to practice. Many felt they lacked sufficient time and support to learn the system, and several did not complete the training. The respondents said that several of their colleagues cried after the training and felt deeply worried about how things would go.

There were many who cried several times during the training week because they felt so incredibly frustrated. (Nurse 6)

#### Unsatisfactory communication

Respondents described a constant and evolving flow of information about the system leading up to Go Live, which made it difficult to keep pace. Despite numerous meetings and forums, the system was not demonstrated before the launch, and the information shared was often general, lacking practical relevance.

There were a lot of videos about the visions with Millennium …, but there was never any practical information about exactly how it would look and how to work with it. That came only after the summer, about one or two months before the implementation, and it was very inadequate. (Administration staff 38)

Communication was experienced as largely one-directional, with little opportunity for dialogue. Questions were met with vague responses or deferred answers, contributing to a sense of frustration. Concerns and feedback raised by the respondents during the pre-implementation phase were often dismissed or met with promises of future resolution.

Respondents noted that the Millennium programme and line organization were perceived as working in silos, and collaboration between workstreams in the programme was lacking; no one had a complete overview. Mixed messages from the central and local levels created confusion and reduced transparency.

It’s been a journey because I’ve felt like I’m here, I’m like it’s just me who doesn’t understand how it all fits together, and then you notice that no one else understands either. (Administration staff 1)

#### Variation in management support

Respondents described varying levels of engagement from first-line managers during the implementation. Some were described as supportive, attentive, and created favourable conditions for preparation, such as pausing other development efforts and allocating time for training. However, some assumed that issues would resolve after Go Live. The pre-launch period involved a heavy workload for first-line managers, particularly around assigning permissions and training, which was challenging to manage.

My boss was very committed and worked really hard to make sure it would be good. She was very understanding about this role as Millennium coach, how it turned out and so on, but it was difficult to be heard at the top. We had good support from each other at the lowest level, but I don’t think we got that much support from above. (Physician 37)

When the respondents began to feel worried, the local managers listened and tried to raise the concerns in the organization. However, the respondents felt that the higher-level management did not listen to or take the concerns seriously.

### During go live

#### A big disappointment

From the start, respondents experienced that there were major problems with roles, assigned permissions and login, which contributed to delays in the registration of care documentation. In addition, information that was transferred from previous systems was difficult to find or was unstructured. For example, return visits and referrals were mixed up.

Several respondents pointed out that Millennium was cumbersome and difficult to use, with many clicks and complicated workflows. Every action took much longer and created uncertainty about whether the task was completed. Millennium was described as illogical, especially regarding medication management and appointments.

Four highly educated people, two district nurses and two doctors, could not manage to prescribe this vaccine. (Nurse 9)

The respondents expressed that functionality that had existed in previous systems could be missing, and new functionality that was promised in Millennium was not delivered. For example, there was no opportunity to prioritize referrals, and the promised functionality to scan medications did not materialize.

You felt cheated; you promised us things that we would get help with, but very few things have worked. (Nurse 19)

Some described feeling that Millennium was unsafe for patients, for example, during medication management, but also from the perspective of information security.

Vital parameters on one patient, i.e. pulse, blood pressure and oxygenation and things like that, appeared in the medical record of another patient. (Physician 16)

However, some examples were perceived as good: for example, the emergency ledger for the emergency room and prescription packages for patients with pneumonia. Some respondents, mainly nursing professionals, also expressed that they viewed the principles of Millennium on real-time documentation and order-based working methods positively.

#### Working in an abstract reality

Several employees experienced stress related to feeling unsure if they had done the right thing in the patient’s record, combined with stress over increased waiting times and whether it could negatively affect the patient. Not being able to perform usual tasks created a sense of inadequacy and doubt about their own professional competence.

An incredible feeling of powerlessness and ethical stress; not being able to do our work because one of our most important tools has disappeared and been replaced with something that does not work. (Physician 8)

The situation was described as participating as guinea pigs in a surreal psychological experiment. It eventually became so absurd that some respondents described that they laughed and cried over the course of time. One respondent described that the patients even apologized for seeking care during Go Live because they felt sorry for the staff.

It was a shock the first day, but okay, this might work; but it pretty soon turned to resignation and cynicism. It felt like we were preparing for some kind of war; that’s why it was completely absurd. (Physician 8)

The poor working environment contributed to the strong emotions that were openly displayed in the workplace during Go Live.

The staff here were not satisfied … they simply cried a couple of times later in the afternoon, so you had to try to calm them down and say that it’s new to all of us and we have to do the best we can. (Administration staff 20)

Some respondents described that they and their co-workers felt so bad that they needed to go on sick leave. Anxiety, dizziness, nausea, sleep problems, heart palpitations, headaches, and panic were common. Their health was affected even outside working hours.

#### Insufficient local support

The respondents described that the information came late, was unstructured, and difficult to find. New working routines were not communicated or were missing. Access to user manuals was unstructured, and the content did not always correspond to reality.

You didn’t have time to read. A lot of mistakes were made because you didn’t understand the user manual or couldn’t find it. (Administration staff 10)

Respondents described receiving great support from colleagues and that collaboration within and between units was strengthened.

When there is a crisis, you always become closer. Everyone had a common goal, and everyone strived to make it work. (Administration staff 23)

Several respondents expressed that the Millennium coaches did a fantastic job. However, for some coaches, the situation became chaotic when many colleagues needed help simultaneously.

We who were coaches, this sounds crazy, but we had to go and hide because we didn’t get a break, …, people were completely crazy and cried, and 20–25 people wanted help at the same time. (Nurse 9)

Floor walkers available in several units did their utmost to be helpful, but they did not know enough about the Swedish healthcare system, the Swedish design of Millennium, or the Swedish language. Respondents also described that the Millennium IT support became overloaded.

#### A minimum of response

Support from first-line managers was described in positive terms in most cases. The managers were attentive and tried to be helpful, but it was difficult because they had no direct knowledge of how Millennium worked. When the problems were highlighted, the respondents felt that they were not listened to. Problems were raised with managers higher up in the ranks, but feedback was usually lacking. Inadequate communication contributed to employees turning to the media with their concerns.

Many of us wrote letters to the newspaper, and it was probably in some way the only way to reach out. (Nurse 9)

### Post implementation

#### Exhaustion and feelings of fatigue

After the decision was made to pause the implementation, some respondents described a strange emptiness and expressed strong fatigue that lingered months later. They experienced fatigue as an obstacle towards changes in general, and digitalization projects specifically. Several respondents described being conscientious and doing their best to make Go Live work. In retrospect, they felt disappointed and sad over the failure. Some even expressed that they felt a little stupid afterwards, manipulated and cheated when expectations were not met. Some felt they had misled others. Respondents still experienced negative consequences in their personal lives; some employees were still on sick leave.

It was a vacuum that lasted for probably two months. Post-COVID has been talked about; here we are talking about post Millennium. (Administration staff 15)

Scepticism developed about the next implementation of EHRs. Many still did not feel ready to gather the strength to start over. Thinking about a new implementation created anxiety and stress. One possible consequence was highlighted; they or colleagues threatened to terminate their employment if Millennium was reinstated.

Many of us will leave our jobs if you even talk about Millennium again. (Nurse 9)

Several of the Millennium coaches were deeply affected and hesitant about taking on similar assignments in the future.

I’ve never pushed myself so hard and set such high expectations on myself, but I’ve probably learned to be careful about what I say yes to without knowing more about what is required. (Administration staff 11)

Some respondents described that their experience as a Millennium coach provided the opportunity for personal development.

I still feel that I have gained a lot of things in my personal development. I think I have learned a lot from it on a personal level. (Midwife 19)

#### Broken trust and lack of accountability

Confidence in higher management and the entire organization was described as destroyed. This was not due primarily to the failure, but rather how the participants felt they were treated throughout the implementation.

It’s been a few months now and I’ve been very, very angry and I still am. Now I’m speaking out but I think they’ve kind of abused their employees. They’ve taken very big risks with their employees’ work environment and health. (Physician 67)

Respondents expressed that there was no apology from higher management for what they had been subjected to regarding Go Live. There was scepticism towards higher management in the region. Trust in the employer was damaged, and confidence in the organization was negatively affected.

We said all the time that this doesn’t work, we don’t know the system, the system doesn’t meet our needs, but still we started. You felt very run over; everyone felt run over. No one has really said that well, we understand or we apologize that this is not happening. no one has given us that security. (Nurse 19)

Respondents expressed that reinsurance was needed from top management that they would listen next time and take what the staff have to say into consideration much earlier.

There were perceived ambiguities regarding the assignments and decision-making mandates between different groups, which created confusion at various levels in the organization. The mandate and responsibility at different management levels in the programme were unclear. Management and governance were fragmented and unsynchronized; cohesive and regional focus was lost.

Respondents specifically expressed that several local managers created opportunities for reflection for employees, which was experienced as valuable after the pause.

We shouted so loudly on this, and it doesn’t feel like anyone listened to us, which was an incredible frustration when we realized that they didn’t care what we said. Trust is very low, unfortunately, and I am not talking about my immediate managers; I am talking about a higher level, those who actually had the mandate to slow this down. (Physician 67)

#### Conditional optimism about future implementations

Several respondents expressed positive feelings about continuing the work on the implementation of a new EHR system. However, most respondents recommended that future implementations should take place on a smaller scale instead of the Big Bang that was made during Go Live.

A small pilot to test how this will be and then adjust routines and guidelines to ensure safety. (Administration staff 21)

The respondents strongly highlighted the importance of a user-friendly EHR system. The functionality should contribute to simplification and to the performance of tasks in an intuitive and efficient way. They also highlighted the importance of proper testing in everyday clinical practice before it is widely deployed.

A future healthcare information system needs to be easy to work in, easy to understand and time-efficient. It should not need so many clicks to get into a medical record. (Nurse 5)

Some respondents believed that there was not enough focus on change management and implementation support. However, others felt that the organization was prepared based on the existing change management plans. Poor foresight, delayed deliveries, late and unstructured information, and short deadlines for planned activities hindered the implementation of the plans and Go Live was therefore not properly prepared for the launch. The training initiatives that were previously described as poor need to be greatly improved in the event of a new Go Live according to the respondents.

Involvement of clinical expertise in the design and implementation was emphasized as important; healthcare professionals know best what is important. In addition, the respondents argued for the importance of standardized working methods and that people should work more equally in the region.

I think there is still some potential in it (Millennium), but it needs a lot for it to work. (Physician 16)

The respondents expressed the importance of management learning from their experiences and using that in future implementations. For example, straight, clear and ongoing information is needed, as well as clear and transparent preparations. The importance of taking earlier research and experiences into account was also stressed. There was a feeling that a new implementation would be an uphill battle, including a lot of resistance from healthcare staff.

Many people probably have a small loss of trust, and it will maybe take some time to build that back. I think the most important thing is probably that people feel that you are trying to really listen to what everyone says and consider how to avoid the same thing again. (Physician 60)

## Discussion

This qualitative study explored end-user experiences of a large-scale implementation of a new EHR system. The findings revealed that participants understood the need for a new system and initially demonstrated strong commitment to the change, with positive beliefs and expectations characterizing the early implementation phase. However, as the Go Live date approached, concerns increased, and confidence in the success of the implementation reduced. The results also highlight insufficient training, unclear task demands within the system, and a lack of communication and feedback. Several participants reported feelings of fatigue and exhaustion and stated that their trust in higher management was damaged, as well as their trust in future implementations. These findings indicate that even though there had been years of preparation and implementation work before Go Live, the degree of organizational readiness had been underestimated regarding organizational, technological and employee preparedness. The findings reveal shortcomings in both individual preparation and structural aspects of the implementation, including feedback mechanisms. From a CFIR perspective, these challenges relate to deficiencies in the inner setting (e.g. readiness for implementation, access to information and resources) as well as the implementation process itself [15]. Furthermore, in line with the NASSS framework [26], the results highlight how insufficient system maturity and limited adaptation to the local context increased complexity across multiple domains, thereby constraining successful adoption. These findings suggest that even a well-planned implementation process is unlikely to succeed if the system is not fully developed or adequately aligned with the organizational context.

According to Weiner et al. [17], a range of undesirable outcomes can occur if there is a lack of readiness: (a) the change effort experiences a false start from which it might or might not recover; (b) the change effort stalls as resistance grows, or (c) the change effort fails.

Respondents acknowledged the necessity for a new information system, citing the obsolescence of the current one and that the functionalities described in Millennium could be beneficial and valuable. Rogers [27] identified relative advantage as the degree to which an innovation is perceived as superior to existing alternatives. When a system is seen to offer clear benefits, individuals are more likely to adopt it. Perceived value reflects users’ belief that the system will positively improve their workflow. The functionality described in the new system echoed core values among employees, an important facilitator for implementation. Weiner et al. [17] emphasized the importance of change valence, how and why members value the change as needed or worthwhile as one determinant for successful change. Individuals committed to the change also display more cooperative behaviour (e.g. volunteering for tasks) and championing behaviour (e.g. promoting implementation) [28]. The results show that in the earlier stages of the implementation, many respondents valued the change as positive; they believed in the collective capability to execute the system implementation and were engaged in the implementation. This changed over time as Go Live approached; the respondents felt stressed and were negative.

Subject matter experts were involved to translate and adapt Millennium to a Swedish context and establish new workflows. Even though many clinicians had been involved in configuration activities as experts, they still lacked an understanding of the whole system. Creating alignment between technology and work practices has been highlighted by earlier researchers [29]. End-user involvement in the design, implementation, and training is critical for success; customizing systems and engaging users improves adoption [2, 17]. As the Go Live date approached, problems and risks were increasingly highlighted in various forums. The absence of feedback on whether these risks had been addressed generated frustration and concern among stakeholders. Despite the number of functions involved, there was a lack of continuous evaluation of progress in terms of employee readiness and system readiness. Real-time collection of feedback is desirable because emerging risks can be solved more proactively rather than reactively. The capture of user feedback and the importance of responding to it promptly have also been highlighted [29].

Compatibility of the system with existing backgrounds, behaviours, and values significantly influences adoption, and Millennium has its origin in a US model of healthcare [27]. Clinicians were expected to participate in team training to learn new workflows; however, the undeveloped system made the training challenging. Our study underscores the need for comprehensive training before, during and after implementation to build confidence, as highlighted previously [16, 21, 22]. The need for end users to get to know the big picture of how the system worked and affected their workflows was clear. However, the incomplete development of the system further complicated the design of efficient workflows, making training in that environment challenging. According to Rogers [27], the compatibility of an innovation with the existing backgrounds, behaviours, and values of its users significantly influences its adoption by potential users.

Respondents highlighted “the value of local support”; for example, access to information about the system’s functionality, local system support and support from local managers. Several studies stress the importance of the leaders’ role in the implementation process and their impact on fostering change [15, 30–32]. Birken et al. [31] highlight the importance of middle managers and suggest they have influence on the implementation of healthcare innovation by diffusing information, synthesizing information, mediating between strategy and day-to-day activities, and selling the implementation of the innovation. Respondents described first-line management in positive terms as attentive and helpful. However, managers had scarce information on how Millennium worked and were thus unable to provide the support their employees needed. The distribution of responsibilities became fragmented with inefficient decision-making and deficiencies in communication.

Only a few studies have examined interrupted EHR implementations. In Kaiser Permanente Hawaii, an EHR system was discontinued and replaced a year after rollout [18]. That study reported organizational conflict and diminished trust in leadership, which improved only after the replacement. Our findings similarly indicate that trust in managerial leadership among healthcare staff was severely eroded and will require substantial effort to rebuild. Participants described critical events that reinforced their perception of managerial betrayal, including the sense that leadership ignored their concerns. This lack of voice and understanding has been highlighted in previous research [18, 21]. Listening to staff during implementation reduces stress related to EHR adoption [33] and should be prioritized. Participants emphasized this as a key lesson from the failure and stressed the need for genuine involvement in future implementations [21, 22].

Respondents described the EHR system as “a big disappointment”, citing inadequate functionality, safety concerns, and usability issues. Transition to EHRs aims to standardize tasks and improve efficiency, but they can disrupt workflows and roles, increase workload, and alter communication patterns [4, 8, 21]. Other reported consequences include productivity loss, cumbersome processes, negative user emotions, and privacy and security concerns [4, 5].

Even successful EHR implementations can have a negative impact on healthcare professionals’ well-being, increasing the risk of errors [22]. Therefore, clear, realistic communication about workflow changes is essential [5]. Participants reported an inability to perform routine tasks due to insufficient training, leading to self-doubt and concerns about patient safety.

Disruptions to work processes can negatively affect employee confidence and morale, leading to stress, frustration, feelings of incompetence, and increased turnover [18, 21, 22]. Exhaustion and complex interfaces contributed to physical symptoms, fatigue, sick leave, and strained personal lives [7]. Negative effects persisted months after Go Live, echoing earlier findings of post implementation challenges [5, 21]. Significant barriers remained regarding digitalization efforts, even months after discontinuation of the EHR system.

Further research is needed, for example, on involvement in the implementation process.

### Limitations

The retrospective approach of data collection may have affected the results. No data were captured during the pre-implementation phase or during Go Live. All data were captured 4 months post implementation. The EHR system was no longer in use when the interviews took place. Maybe the results would have been different if the data had been collected during each phase? However, when the respondents talked about the whole implementation process, they could relate each phase to another and thereby give a nuanced picture of their experiences over time.

A limitation of this study is that not all personnel involved were interviewed; the focus was on staff from the sites where the Go Live of Millennium was carried out. Several other professionals were involved in the implementation, and their experience could have added other perspectives to the study; for example, members of the programme team or other roles with responsibility for the implementation.

This is a unique case because the implementation stopped after a few days, yet the insights and lessons can be of value for other healthcare systems planning large-scale EHR implementations. However, the local context is always different and affects the outcome, which may limit transferability.

The researchers’ experience in healthcare as practitioners, management and in operations development and within digitalization was seen as a reflective access in the analysis and not as bias as described by Braun and Clarke [25]. The number of interviewers could have been a limitation; however, saturation was reached in the analysis, which could validate that data was collected in the same manner. Also, the fact that only the authors of this paper analysed the data can strengthen the results.

The material from the 73 interviews is substantial and represents an important contribution. Further analysis from multiple perspectives could be conducted, given the extensive dataset and the limitations of the scope of this article; for example, on co-creation, involvement and emotional effects. Further research could also follow up on the long-term effects, for example, 2–5 years after large-scale implementation of a mega system.

## Conclusions

This study described the challenges of a large-scale implementation that failed. The findings add insights that strengthen earlier research according to the field of implementation science.

First, to accomplish readiness, the end users highlighted that they need to be involved in the implementation process and understand the whole picture, both the object being implemented as well as new working routines, with opportunities to become familiar with them.

Second, dialogue and feedback between co-workers and top management are crucial for readiness on an organizational level. Participants experienced that a lack of dialogue can demolish trust and negatively affect continual implementation.

Third, participants described that incomplete readiness during implementation can contribute to anxiety, feelings of abandonment and being in an abstract reality, which also negatively affect confidence in the professional roles.

The system was presented as a modern, comprehensive solution expected to meet a wide range of needs, which raised expectations. When the outcome failed to meet these expectations, it resulted in a sense of emptiness and disappointment among the participants, echoing the tale of the emperor’s new clothes.

## Supplementary Information

Below is the link to the electronic supplementary material.

Supplementary Material 1

## Data Availability

The datasets presented in this article are not readily available in accordance with federal requirements and standards and guidelines for the protection of participants’ privacy and to maintain confidentiality. Requests to access the datasets should be directed to Dr. Susanne Gustavsson (susanne.m.gustavsson@vgregion.se).

## Abbreviations

EHR: Electronic health record
RTA: Reflexive thematic analysis
VGR: Region Västra Götaland
