# Supplementary material for: “The emperor’s new clothes”: a qualitative study of end users’ experiences from a failed large-scale implementation of an electronic health record system
Source: BMC Health Serv Res. 2026 Jun 23;26:869. doi: 10.1186/s12913-026-14991-4 (PMC13295468; doi:10.1186/s12913-026-14991-4)
Supplement: Supplementary file 1 — Supplementary Material 1 [file 12913_2026_14991_MOESM1_ESM.docx]

**Appendix 1**

**Background Questions**

1. Where do you work? How long have you worked there? Professional category? Age? Gender?
2. How digital are you?

A. “Mostly at work” = I mainly use digital technology at work and also in everyday life for reading, entertainment, banking, etc. I try to put technology aside when I’m off work.

B. “Full-time consumer” = Digital technology is used both at work and in leisure time. I’m a practical consumer with broad usage, e.g., banking, shopping, keeping in touch with friends, and entertainment. I have no problem putting technology aside when I want to.

C. “All-in” = Digital technology is used regularly for administration (banking, booking tickets, etc.), shopping, social contact, and entertainment. I also use technology to make my voice heard, post content, join discussions, and acquire new knowledge. It’s almost hard to put it aside because it permeates my daily life.

D. “Almost never outside work”

**Preparations before go-live (think about roughly one year back)**

1. What role did you have during the Millennium implementation?
2. How do you perceive the need for a new information system?
3. What were your expectations of Millennium?
4. How did you experience the information you received during the implementation process?
   1. About the implementation process: what would happen when, with whom, etc.?
   2. About the Millennium system: how did you receive information?
   3. About new workflows resulting from working in Millennium.
5. Can you tell us about the training you received before go-live? How prepared did you feel to use Millennium after the training?
6. How did you experience the preparations leading up to Millennium’s go-live?
   1. What preparations were made in your ward?
   2. How did these preparations affect your daily routines and workflows?
   3. Did you collaborate with other wards/functions to adapt new workflows? If so, how?
   4. What support did you receive in your unit before go-live? From whom and in what way?
7. Were there any other change initiatives in your ward at the same time that could have affected the planning/preparations for go-live? How did these affect the process?

**Go-live and working in the system**

1. How did you experience working in the system? Which parts of Millennium did you use most daily? Please share specific situations. Challenges and opportunities?
2. What support was available during go-live?
   1. If you needed help with Millennium – whom did you ask? Did you use any form of support, Millennium coaches, other?
   2. Did you receive any additional training after go-live? If yes, what kind?
3. How did you experience collaboration with support functions during go-live? Would you have wanted any additional support, and if so, what?
4. How did you experience collaboration with colleagues during go-live?
5. What was done to facilitate go-live in your ward?

**Future**

1. In what way has the implementation process affected you as an employee?
2. What would be needed for you to feel confident in future planning for implementing new healthcare information systems?
3. What can we learn from what happened that caused the go-live to be aborted?
4. When a new healthcare information system is to be implemented in the future, how can the implementation process be improved? (Information, training, practice, support)
5. Do you have any other experiences/lessons learned you would like to share?
